# Free Will as Defined by the Constrained Disorder Principle: a Restricted, Mandatory, Personalized, Regulated Process for Decision-Making

**DOI:** 10.1007/s12124-024-09853-9

**Published:** 2024-06-20

**Authors:** Yaron Ilan

**Affiliations:** Faculty of Medicine, Hebrew University and Department of Medicine, Hadassah Medical Center, Jerusalem, Israel

**Keywords:** Artificial intelligence, Variability, Complex systems, Free will, Decision making

## Abstract

The concept of free will has challenged physicists, biologists, philosophers, and other professionals for decades. The constrained disorder principle (CDP) is a fundamental law that defines systems according to their inherent variability. It provides mechanisms for adapting to dynamic environments. This work examines the CDP's perspective of free will concerning various free will theories. Per the CDP, systems lack intentions, and the "freedom" to select and act is built into their design. The "freedom" is embedded within the response range determined by the boundaries of the systems' variability. This built-in and self-generating mechanism enables systems to cope with perturbations. According to the CDP, neither dualism nor an unknown metaphysical apparatus dictates choices. Brain variability facilitates cognitive adaptation to complex, unpredictable situations across various environments. Human behaviors and decisions reflect an underlying physical variability in the brain and other organs for dealing with unpredictable noises. Choices are not predetermined but reflect the ongoing adaptation processes to dynamic prssu½res. Malfunctions and disease states are characterized by inappropriate variability, reflecting an inability to respond adequately to perturbations. Incorporating CDP-based interventions can overcome malfunctions and disease states and improve decision processes. CDP-based second-generation artificial intelligence platforms improve interventions and are being evaluated to augment personal development, wellness, and health.

## Introduction

Free will is a complex philosophical concept that has intrigued thinkers across many disciplines (Menges, 2022). There is a wide range of views regarding free will among physicists, depending on personal beliefs, philosophical preferences, and the interpretation of scientific principles (Berniūnas et al., 2021). The constrained disorder principle (CDP) is a fundamental law that defines systems according to their inherent variability. It provides mechanisms for adjusting to dynamic environments (Ilan, 2022a).

This paper examines the CDP's perspective of free will concerning various free will theories and highlights the possibilities of using CDP-based platforms to enhance system functionality, implying its effectiveness.

### Free Will in the Universe

An individual's free will refers to the capacity to choose between different possible courses of action unimpeded (Lavazza, 2016). Humans are capable of making choices and determining their fates. Despite reasons to believe that numerous factors influence a person's free will, there is no scientific consensus against free will. The concept of "free will" often refers to conscious decisions that influence a person's life, even if innate dispositions, external environments, or randomness shape those decisions (Baker, 2007; Bonn, 2013; Klemm, 2010). It can be argued that unconscious processes can be considered part of an individual's will, even when they help determine their conscious behavior (Bonn, 2013; Klemm, 2010).

In behavioral science, genetics, and the environment influence people's behavioral tendencies in ways beyond their control (Baker, 2007). It is assumed that a person is constrained in the range of decisions and behaviors they are inclined to make or contemplate in any situation (Baer et al., 2008; Nicholas, 2014). Studies of the neuronal circuitry suggest that conscious choices are not genuinely free (Lavazza, 2016; Liljenström, 2022; Maoz et al., 2019).

A person who believes in free will tends to favor internal instead of external explanations for another's behavior (St Quinton et al., 2023). It was argued that moral systems would collapse if everyone did not think they were accountable for their actions—and thus deserved reward or punishment (Björnsson & Persson, 2012). It implies that it is valuable to maintain confidence in free will, even if it is an illusion. Others claim morality can exist without free will or that free will contributes to intolerance and revenge-seeking (Brembs, 2011; Caspar et al., 2017; Danaher, 2022). Another challenge is defining free will for non-human living systems and non-living systems.

### The CDP: a Universal Law for All Systems in Nature

According to the constrained disorder principle (CDP), all systems in the universe are defined by their inherent variability (Ilan, 2022a). The CDP states that inherent variability is required for proper function, enabling continuous adaptation to ever-changing internal and external environments. The CDP is schematically formulated using the B = F formula, where *B* stands for the boundaries of variability and *F* for system functionality and efficiency (Sigawi et al., 2023). The boundaries determine the degree of variability by maintaining a desired range to ensure adaptability to dynamic pressures. All living and non-living systems respond to their noisy environments by enabling more variability or constraining the degree of variability required for optimizing their function. The proper functioning of systems implies boundaries that respond adequately to fluctuations in environmental noise, while system dysfunction results from malfunctioning boundaries (Ilan, 2023a, 2023b).

Per the CDP, randomness, and disorder are fundamental to all systems throughout the universe, and their interactions direct their development (El-Haj et al., 2019; Forkosh et al., 2020; Ilan, 2019a, 2019b, 2019c, 2019d, 2020a, 2020b, 2022, 2022c; Ilan, 2023; Ilan, 2019; Ilan-Ber & Ilan, 2019; Shabat et al., 2021). Physical, chemical, and biological systems exhibit this property (Ilan, 2019a, 2020a, 2020b; Ilan 2019). It states that variability exists at the quantum level and above. Variability exists throughout living systems in genes, molecules, proteins, processes, and cellular and organ functions (Boripuntakul et al., 2022; Bosch et al., 2021; Chiera et al., 2020; Crawford et al., 2022; Finn & Misteli, 2019; Forte et al., 2019; Genon et al., 2022; Kirschner & Mitchison, 1986; Mitchison & Kirschner, 1984; Saha & Baumert, 2019; Schutte et al., 2022). The CDP argues that the degree of variability, which reflects the boundary function, distinguishes between subjects and explains their phenotypes (Ilan, 2023b; Sigawi et al., 2023).

Variation in specific genes modulates behavior, enabling the identification of emerging patterns. Molecular variation plays a role in shaping behavior and its sources and types (Andersen & Rockman, 2022; Niepoth & Bendesky, 2020). Genetic segregation refers to the separation of genetic variants into reproductive cells. Statistically, segregation analysis determines whether a significant gene for the phenotype can explain phenotypic patterns within families (Gupta et al., 2023; Hallatschek et al., 2007). Behavior is shaped by genetic variation, drift, and selection at intergenerational timescales. The effects of variation vary at different levels—in sensory perception, internal neuronal circuits, and outside the nervous system. The nervous system can respond acutely and adapt behaviorally to environmental changes at multiple timescales, ranging from milliseconds to years (Niepoth & Bendesky, 2020). In dynamic environments, measuring behavior consistently in individuals and comparing it across individuals is challenging.

### Per the CDP, Systems Regulate Their Degree of Variability in Response to Perturbations, and There is No Free Will

According to the CDP, free will does not exist in the sense of a choice based on some undefined top-down or bottom-up mechanisms. No "wills" or "desires" exist in atoms, molecules, or organs. All living and non-living systems function as machines using this mechanism to adapt to a dynamic world (Ilan, 2023).

Systems are characterized by a degree of variability necessary for their function and energy conservation (Ilan, 2022c). Living systems differ from nonliving systems in their range of variability, with living systems having a more comprehensive range than non-living systems. Systems' boundaries adjust the degree of variability independently of a will (Ilan, 2022a, 2023, 2023). However, boundaries allow systems to "choose" how to respond to ongoing pressures in their environments. Rather than "selecting" in the conventional sense, it is about how a system is designed. Regulating variability is essential to optimizing a system's functionality (Sigawi et al., 2023).

According to the CDP, memory and learning do not exist. Instead, how the boundaries react to pressures determines system behavior. Identical systems may behave the same way. Nevertheless, their differences are reflected in their altered boundaries of variability due to past events that changed them, allowing for a lesser or greater degree of variability. Per the CDP, system evolution does not take place along temporal trajectories. Evolution is a result of continuous perturbations and pressures between systems. A system's functionality depends on its ability to adapt to changes by its dynamic boundaries. It does not necessarily reflect improvement but is a means for optimizing function under varying conditions (Ilan, 2023, 2023, 2023b; Sigawi et al., 2023). Table 1 describes some of the notions of the CDP on free will.

**Table 1:** The Constrained Disorder Principle's (CDP) perception of free will

The CDP	• The CDP defines systems by their inherent variability, mandatory for proper functioning • The dynamic boundaries of the variability provide mechanisms for adjusting to environmental perturbations • All systems respond to their noisy environments by enabling more or less variability to optimize their function • Optimal performance occurs "at the edge of chaos."
Free will	i. "Free will" is embedded within the variability range determined by the boundaries that adapt to pressures ii. It is inherently conditioned, and "freedom" is inherent to the systems' design
Intentions	• Systems are not intentional. Atoms and organs do not desire • The CDP lacks the personification of systems
Choices and selections	• Freedom to select and act is assembled into a systems design with a built-in and self-generating mechanism for coping with noise • Choices are not entirely predetermined but reflect ongoing adaptation processes to pressures
Decisions	• The function of the brain and neurons is unpredictable within constraints, which are reflected in brain decisions • When a subject is "in control of a decision," it does not mean freedom to choose but reflects a yet-to-be-discovered variability-based machinery designed to handle internal and external noises to guide the selection process
Evolution	• System evolution • does not take place along temporal trajectories and does not imply improvement • Randomness and disorder are fundamental to all systems, and their interactions direct their development
Dualism and trajectories	• Neither dualism nor a metaphysical apparatus dictates choices • There are no top-down or bottom-up trajectories
Determinism and pre-determinism	• Randomness controls functionality and is essential for proper function • Prediction is impossible due to the dynamic nature of the noisy universe • The CDP is not entirely deterministic, providing systems with inherent freedom • The process is not wholly pre-determined since everything changes constantly and dynamically
Causality and goals	• Systems are designed to function efficiently and do not follow a time course to reach a future goal • Their only objective is to function by handling noises • Systems are not concerned with their future • Causality is inherent in how systems are designed. There is no direct linkage between options and outcomes
Consciousness and creativity	• CDP relates to physical apparatuses. The lack of knowledge of the mechanism behind the mind does not rule out the existence of such apparatuses • Brain variability facilitates cognitive adaptation to complex, unpredictable situations • Mental states reflect neuronal inherent variabilities • Creativity reflects flexibility under novel situations
Human behavior	• Human behavior is a reflection of machinery that dictates responses based on variability • As a collection of molecules and organs, humans behave under the same principle of constrained disorder • Human actions reflect an underlying physical variability in the brain and other organs for dealing with unpredictable noises
Diseases	• Disease states are characterized by inappropriate variability, either too low or too high, which results in an inability to respond adequately • A lack of adaptation to perturbations results in difficulties, unwanted feelings, and responses
Correcting malfunctions	• Incorporating the CDP into interventions can improve decision processes and overcome disease states • Platforms based on the CDP can enhance personal development, wellness, aging, and mental health

### Theories of Free Will Through the Perspective of the CDP

Free will is described differently by numerous philosophical views. This section describes several of these theories from the perspective of the CDP.

i. Determinism suggests only one possible outcome incompatible with free will (List, 2014). Classical determinism holds that prior events and natural laws determine every event. These deterministic laws govern the universe, including human actions predetermined by initial conditions (Santo & Gisin, 2023; Strien, 2021). Free will does not exist in determinism because determinism is accurate and incompatible (Balaguer, 2018).

The CDP platform provides machinery with an inherent degree of freedom. It does not support a will to select. However, it possesses a sophisticated automatic selection mechanism embedded in every system's design. The process is not entirely predetermined since everything changes constantly and dynamically (Ilan, 2023b; Sigawi et al., xxxx).

Determinism is divided into causal, logical, and theological types (Dotsenko & Pchelina, 2021). A causal determinist believes everything has a prior cause, making any other possibility impossible (Baumeister et al., 2022). In addition to past and present events, natural laws determine future events. Logic determinism holds that all past, present, or future propositions are true or false (Prior & Prior, 1967; Schumann, 2021). Hence, the problem of free will relates to how decisions are made with freedom, given that the present determines the future as true or false. Free will face a unique problem since recommendations about the future are already confirmed in the present, referred to as the problem of future contingents (Bradley, 1959; Gunji et al., 2020; Rummens & Cuypers, 2010).

Theological determinism infers that all events are preordained by a monotheistic deity or destined to transpire because of its omniscience (Wisniewski et al., 2019). Omniscience is knowing everything to be known, including future events. It is associated with a creator deity (Kraal et al., 2013). As a form of high-level predeterminism, it implies the existence of destiny or predestination. All events and outcomes are determined in advance. Free will does not exist, and God controls a person's actions. Theological determinism relies on divine foreknowledge. Due to God's perfect omniscience, everything he knows about the future will inevitably happen, so it is already predetermined. The antecedent conditions are the nature and will of God. Free will is compatible or incompatible with weak theological determinism based on one's philosophy of omniscience. As long as God knows humans' actions before they happen, theological determinism claims that he does not affect the outcome. Theological fatalists assume that omniscience and free will are incompatible, while soft theological determinists argue that omniscience does not entail that God affects the outcome, meaning that free will and omniscience coexist. The providence of God is "compatible" with voluntary choice (Byerly, 2017; Free & Will, 2023; Gittinger, 2022).

CDP has the following perspective on the relationship between the past and future. Evolution is a process of continuous changes in inherent variability; however, it is not along a temporal trajectory. Systems continuously adapt to external pressures without an apparatus for learning from the past that determines the future. Systems adjust their inherent variability by their variability borders to adapt to continuously changing pressures. CDP does not follow a top-down or bottom-up approach. Thus, current events may evolve from past events but do not directly relate to them. Systems are designed with an inherent mechanism that continuously selects the range of their inherent variability (Ilan, 2023b).

Per the CDP, disorder exists in every biological system. It implies that variability is necessary for systems to function correctly. Disorders enable continuous adaptation to pressures, resulting in optimal functions. An inappropriate decrease or increase in the variability leads to malfunctions. It is not entirely deterministic and lacks freedom of choice (Ilan, 2022a, 2023b).

ii. Incompatibilism holds that free will is incompatible with determinism (Wisniewski et al., 2019; Zürcher et al., 2019). If the world is deterministic, the feeling of freedom to choose an action is an illusion. Free will and determinism are logically incompatible, and whether people have free will boils down to whether or not their actions are determined (Vihvelin & Vihvelin, 2013).

Incompatibilists fall into camps. As outlined above, determinists believe determinism is true and free will is false. However, contrary to this, libertarians contend that determinism is false; thus, free will is true (Pleasants, 2019).

According to metaphysical libertarianism, determinism is false; therefore, free will is possible. It is associated with non-materialist viewpoints, including dualism and models of consciously vetoing actions or competing desires (Hartman, 2020; Miles, 2013).

Unlike physicalism, metaphysical libertarianism requires physical indeterminism, such as probabilistic subatomic particle behavior (Miltenburg & Ometto, 2019; Potter & Mitchell, 2022). Libertarianism is divided into non-physical theories and physical theories (Iyer et al., 2012). Non-physical theories claim that physics cannot fully explain brain events that lead to actions, so the world cannot be closed by physics (Owen, 2020). In interactionist dualism, a will, mind, or soul override physical causality (Pitts, 2020). A libertarian view of free will cannot be reconciled with physical determinism, which implies only one possible future (Wisniewski et al., 2019).

According to the CDP, degrees of freedom are reflected in systems' variability boundaries. A system does not have a veto power. It is capable of adapting to pressures. Neither dualism nor an unknown metaphysical apparatus dictates choices (Ilan, 2022a). Systems are designed with inherent variability, which changes in response to perturbations. It is physical since system parts are designed to perform such a function. Subjects' choices cannot be their own, even if they influence their lower-level physical systems, as with libertarian free will (Culler, 2004; Farhadi, 2023; Leftow, 2009).

Incompatibilist theories are categorized according to the type of indeterminism they require, including uncaused events, causally non-deterministic events, and agent/substance-caused events (Ekstrom, 2019). Non-causal accounts imply that free will is not attributed to an agent or a physical event (Lavazza, 2019; Pleasants, 2019). Every intentional action requires a choice or volition - a willing, trying, or endeavoring on the agent's part. Deliberate actions are interpreted as free actions (Brown, 1989; Michaelsen & Esch, 2023). Rather than analyzing the agent's causation in terms of mental states or events, such as desire, belief, or intention, it is considered a matter of spontaneity and creativity (Beaudoin et al., 2019; Bonicalzi & Haggard, 2019). A person's intention does not determine the freedom of a given action. Intentional actions are self-generating. Activating someone's brain can result in actions without any relevant desire or intention from that person (Lumer, 2018). Physicalist mental models underlie event-causal accounts of incompatibilism (Wisniewski et al., 2019). The agent causes certain indeterministic events. Event-causal theories include deliberative indeterminism, centered theories, and efforts of will theory. The first two imply that free will does not have to be a fundamental aspect of the universe, while ordinary randomness provides the necessary "elbow room" (Ekstrom, 2019; Kane, 2006).

Deliberate indeterminism implies that indeterminism occurs earlier in the decision process (McCall & Lowe, 2005). An indeterminate set of possibilities is provided without introducing the risk of random decision-making or luck into the decision process. Deterministic selection processes rely on earlier preferences established by the same process. A centered account proposes that when choosing between two options, the strength of reason is considered, although there is still a probability that the weaker candidate is chosen. The effort of will examines how willpower affects decision-making. It suggests that agent volition processes are indeterminate like physical events are indeterminate - and that the agent leads to outcomes (Aguilar & Sommaruga, 2020; Ainslie, 2020; Gluth et al., 2020; Manzini & Mariotti, 2014; O'Connor, 1993). The agent assumed the power to intervene in the physical world. Prior events do not causally determine an agent's actions. It is difficult to determine what led an agent to make a particular choice, which suggests that the choice was based on luck rather than free will (Elzein, 2023; Griffith, 2010; Vihvelin & Vihvelin, 2013).

Free will skeptics argue that free will is incompatible with both determinism, as caused actions are not free, and indeterminism, as random actions are not free (Vargas, 2004).

According to the CDP, "intentions" are reflected in boundaries that respond to pressures. It defines a built-in and self-generating mechanism, which can be viewed as "freedom." However, it lacks intention and does not involve causation (Ilan, 2022a, 2023b). Randomness is fundamental to all natural systems and is essential to proper function if regulated, allowing it to adapt to pressures (Ilan, 2023; Ilan, 2023; Sigawi et al., 2023). The CDP lacks the personification of systems. Biological systems lack desires and intentions and are designed to function. Their inherent variability will enable them to survive and optimize functionality under random environments. The function must involve “random decision-making." Optimal performance occurs "at the edge of chaos." (Ilan, 2022a, 2023b) The universe has a mechanism that permits all systems to interact and adapt. A system's boundaries must function correctly to keep efficiency and functionality. The optimized efficiency is achieved when the variability boundaries allow proper pressure adaptation. Causality is inherent in systems' design but lacks a direct linkage between options and outcomes (Sigawi et al., 2023).

iii. Compatibilists claim that free will can coexist with determinism (Lavazza, 2016). Determinism is essential for free will, as choices entail a preference for one action over another. Individuals can make meaningful choices despite the universe operating according to specific laws and be responsible for their actions. Rather than focusing on the absence of deterministic influences, they emphasize the nature of choices. Compatibilists underscore the importance of understanding free will in making meaningful decisions rather than being free from all influences (List, 2014; Rogers, 2004; Uncompromising & Incompatibilism, 2010). The standard solution for philosophers to the gap between our sense and existing scientific knowledge is to argue for the existence of "compatibilist freedom." Subjects act according to their values, but this freedom does not refute scientific determinism. Every action results from the nervous system, and the sum of the events in a subject's life is thus deterministic. However, when a decision is made per a person's scale of values, it is considered free (Honderich & Honderich, 1990; Klemm, 2010; Lau & Hiemisch, 2017). Classical compatibilists define free will as freedom of action, which means that if one counterfactually decided to act differently, one could have done so without physical impediment (List, 2023). The dilemma is resolved by arguing that humans have free will if they are not externally constrained or coerced. Rather than identifying free will as a psychological capacity, compatibilists define it as the ability to direct one's behavior based on reason. Freedom of choice is separated from freedom to enact it by modern compatibilists (Pleasants, 2019).

The CDP views the machinery as automatic. Choices are not predetermined but reflect ongoing adaptation processes to dynamic pressures. There is no freedom of decision-based on intentions but rather on a built-in mechanism of coping with noise by incorporating part of it as required for optimizing functions (Ilan, 2023, 2023). Throughout the body, all processes, from atoms to whole organs, are governed by the same law. It permits freedom within the laws of nature (Ilan, 2023b).

iv. Causality and free will: Free will conflicts with natural law when causal closure or physical determinism is asserted (Lavazza, 2016; Front matter, 2020). that preceding events entirely determine the future, Physical determinism asserts and causal closure asserts that physical events do not have causes outside the physical domain (Bishop, 2006). Incompatibilists reject the notion that free action consists of "voluntary behavior." According to them, free will implies that an individual is responsible for his actions (Timpe, 2007). According to compatibilists, mental reality is not necessarily causally efficacious, and freedom of will concerns rational decisions. As a result of causal determinism, events within a paradigm are bound by causality, and prior conditions entirely determine any state of an object or event (D’Ariano, 2018; Zürcher et al., 2019^).^ In nomological determinism, or scientific determinism, the past and present dictate the future entirely because of rigid laws, and every event is inevitable because of previous events (Oh et al., 2023; Okabe et al., 2009). Quantum mechanics challenges this view.

According to predeterminism, all events are determined in advance (Lavazza, 2016; Rappaport, 2011). A divine being, fate, or some other force is responsible for all occurrences in history, past, present, and future, including human actions (Solomon, 2003). The universe has a fixed natural order, and human actions cannot change the outcome (Clark et al., 2019; Kronfeldner, 2009; Laurin et al., 2011; Waggoner & Uller, 2015). Fate implies a predetermined course from which one cannot deviate and has no control (Chen, 2023; Waggoner & Uller, 2015; Zhang, 2021). Although fate is related to determinism, it does not explicitly claim physical determinism. Destiny implies that a course cannot be deviated from and does not claim responsibility for setting it. The mechanism by which that destined outcome is chosen could be free will (Bargdill, 2006; Hallett, 2016).

The CDP does not consider causality, which implies continuous attempts for improvement. Systems are designed to function under noise but not to improve over time. Changes reflect the dynamic boundaries that continuously respond to perturbations. As a collection of molecules and organs, humans behave under the same principle of constrained disorder (Ilan, 2023a, 2023b). Each process is subject to inherent variability as it interacts with its noisy internal and external environments. Biological variabilities are similar to all other systems and include genetic, transcriptional, biochemical, cardiac, blood pressure, gait, and neuronal variations (Boripuntakul et al., 2022; Bosch et al., 2021; Chiera et al., 2020; Crawford et al., 2022; Finn & Misteli, 2019; Forte et al., 2019; Genon et al., 2022; Kirschner & Mitchison, 1986; Mitchison & Kirschner, 1984; Saha & Baumert, 2019; Schutte et al., 2022). It is a regulated control because a mechanism constantly manages pressures; however, it does not imply a predetermined destiny or fate. Systems can deviate from their path at any time. The dynamic boundaries deal with unpredictable, endless noises that allow systems to function (Sigawi et al., 2023). Systems are designed to function efficiently; however, they do not follow a time course to reach a future goal (Ilan, 2022a). The CDP prohibits top-down processes from dictating functions. The solitary goal is to continue functioning efficiently by handling the noises while not defining the future (Ilan, 2023b).

xxii. Free will is one aspect of the mind–body challenge, which is about the relationship between the mind, which includes consciousness, memory, and judgment, and the body, such as the brain and nervous system (Lavazza, 2016). According to Cartesian dualism, the mind is a non-physical substance containing consciousness and intelligence and is not identical to the brain and body. Despite their interaction, both worlds retain some autonomy (Christofidou, 2018). The external mind controls bodily actions. An interpretation of incompatibilist free will is that the external mind controls physical events rather than the physical world being deterministic (Pleasants, 2019). A dualistic interactionist accepts some physical events as a result of mental acts, while some mental acts can result in physical events (Moini et al., 2024). According to physicalism, everything exists based on its physical properties (Introduction, 2018). It implies that non-physical substances like independent minds do not exist. Reductive physicalism holds that all things can be reduced to their physical basis. Non-reductive physicalism asserts that mental properties are distinct from physical properties, implying that mental states are not reducible to physical states (Brakel, 2021). Even though mental states may differ from neurological states, it does not rule out the possibility of mental states being correlated with neurological states. The anomalous monism theory correlates mental and physical properties, implying causal reducibility (Yalowitz, 1998).

The CDP applies to neurological functions but does not apply to the mind or consciousness. Based on this principle and the current knowledge, conclusions about the mechanisms underpinning the "mind" cannot be made. Nevertheless, humans operate according to the CDP, dealing with unpredictable noises to cope with pressures in their daily lives (Ilan, 2023, 2023, 2023b).

Incompatibilism differentiates the mental and physical, pointing out the incompatibility between determined physical reality and one's subjective will experience (Pleasants, 2019). According to metaphysical libertarians, free will must assert influence on physical reality. The mind exerts influence instead of ordinary system randomness and must be distinct from the body to achieve this (Farhadi, 2023). According to compatibilists, freedom can exist for reasons unrelated to metaphysics, and deterministic relationships exist in the physical world, including the brain (Sie & Wouters, 2010; Wisniewski et al., 2019). Cognitive naturalism is based on the idea that the mind is part of nature, possibly merely a component of many complex self-programming feedback systems and neural networks (Murphy et al., 2007). In non-naturalism, such as mind-body dualism, the human mind exists independently of its body. It includes traditional religious metaphysics and less common compatibilist concepts, such as perceiving, thinking, and choosing freely (Burkhardt & Imaguire, 2002; Roskies, 2006).

Buddhism recognizes freedom and determinism but rejects the notion of a total external agent despite its focus on human agency (Javanaud, 2018). Buddhists do not believe in absolute free will or determinism. There is a middle doctrine called "dependent origination," "dependent arising," or "conditioned genesis." Because ignorance is the root of every volition, every action is conditioned. It asserts that free will is inherently conditioned and not genuinely free. Karma refers to the cause and effect of moral actions (Diller & Lattal, 2008; E S & Anacaryiako D., 2022). In Hinduism, karma determines one's fate in future lives (Hutchinson & Sharp, 2008). The Buddhist concept of absolute freedom of choice is considered unwise because it ignores physical needs and circumstances. It is also incorrect to suggest that humans have no choice in life or that their lives are predetermined (Religion, Philosophy and Culture, 1995).

Physics still has not resolved the complex problem of consciousness and the interaction between conscious and unconscious brain activities (Meng, 2024).

The CDP acknowledges our inadequate knowledge about the machinery of the mind that does not rule out the existence of a physical apparatus behind it. Per the CDP, mental states are caused by neuronal functions with inherent variabilities. The CDP implies that "freedom" is inherently conditioned and not entirely free, and systems are not concerned with their future (Ilan, 2022a, 2023a, 2023b; Sigawi et al., 2023).

vi. Freedom of action is not synonymous with free will. A person's freedom of choice is distinct from the freedom to implement that choice, i.e., to carry out one's choice (Schwartz & Cheek, 2017). Some argue that free will is simply choosing what one can do based on constraints. A coerced agent can make free choices if the coercion coincides with the agent's intentions and desires (Zürcher et al., 2019). Most "classical compatibilists" believe that someone acts on his own will when he desires to do the act; however, it is also possible for the person to act otherwise if he has decided to (Shatz, 1988). In the absence of God, an infinitely powerful demon, and other such possibilities, free will can be viewed as unpredictability (León Serrano, 2018). The knowledge of the current state of the world is limited by chaos and epistemic limits, making the future uncertain. The only thing that can be defined clearly is "expectations,” enabling us to do "otherwise" rather than an unknown and unknowable future (Cook & Wagenaar, 2012; Maciag, 2022).

According to the CDP, functions reflect the automatic selection of a will and an act. "Freedom" to select and act is built into the design of systems. There is no consideration of "desires" or "intentions." A lack of knowledge does not cause chaos; instead, a degree of variability is an integral part of systems and essential for proper functioning. System optimization occurs when systems are at the edge of chaos (Ilan, 2023a, 2023b; Sigawi et al., 2023).

### The CDP's Perspective on the Science of Free Will:

I. Physics and quantum physics: No single law of physics can explain all system functions. According to early scientific thinking, the universe is deterministic (Strien, 2021). In modern science, there is a mixture of deterministic and stochastic theories. There is inherent indeterminacy at the quantum level, and quantum mechanics disputes physical determinism, preserving an element of unpredictability in the universe (Maar, 2019). Some physicists argue that this inherent indeterminacy could allow for free will. Although the uncertainty principle does not necessarily represent intrinsic indeterminism, it represents limits in measurement precision. Chaos theory emphasizes the effects of such microscopic events; however, the relevance of such prospective indeterminate activity to free will is contested (Landsman, 2020).

The CDP accounts for the quantum world without distinguishing it from the macroscopic world. Variability characterizes all systems, and the difference between them lies in their variability range. “Freedom” is inherent to how systems are designed, reflecting the degree of the universe's unpredictability (Ilan, 2023a; Sigawi et al., 2023).

The laws of quantum mechanics are probabilistic rather than deterministic (Klein, 2020; Vaidman, 2014). Einstein believed there had to be a hidden layer below the quantum level, so if we could uncover this hidden layer, we could replace quantum mechanics' probabilistic laws with deterministic ones (Mehta et al., 2019). In Heisenberg's and Bohr's view, nature's fundamental laws are probabilistic. As a result, previous events do not deterministically trigger some physical events, and some happen (Tarozzi & Macchia, 2023).

Per the CDP, each system is defined by its degree of variability; its function is not entirely deterministic and has a degree of freedom to adapt to the surroundings dictated by the variability boundaries (Ilan, 2022a, 2023b). Hence, it is similar to Heisenberg's and Bohr's views that variability is inherent. Per the CDP, this unpredictability is mandatory for proper function (Ilan, 2022a).

Quantum mechanics claims that events can be predicted only by probabilities, raising doubts about whether the universe is deterministic (Strien, 2021; Vaidman, 2014). The environment and random genetic mutations steered evolution as it progressed. Even though they arise from more fine-grained phenomena, these "forces" are causally autonomous (Hershberg, 2015; Cvijović et al., 2015; Seed et al., 2012; Tomasello, 2022). A quantum indeterministic model introduces random occurrences at the atomic and subatomic levels. Mental processes cannot affect probabilistic outcomes like volition when a person's actions are based on complete quantum randomness (Ebert & Wegner, 2011; Ekstrom, 2003; Title, 2021). These events can demonstrate incompatibilist free will if their apparent indeterminacy reflects the underlying indeterminacy of the physical construct (Caro & Putnam, 2020).

According to the CDP, systems do not change along an evolution vector (Ilan, 2023b). CDP describes an adaptive platform for coping with pressures that determine the outcome. Elephants are thought to be descended from the semi-aquatic hippo-like Moeritherium that lived 35 million years ago (Deraniyagala, 2020; Liu et al., 2008). It does not imply that the Moeritherium is a "lesser" animal. In the CDP, evolution responds to environmental changes and does not infer improvement. Systems in the universe are machines that function, and their sole "desire" is to function in noisy environments. CDP defines the mechanism for doing so. Processes are not temporal events aimed at a purpose and do not reflect causality (Ilan, 2023b; Kane, 2006).

Fractal geometry and chaos theory deal with complex, dynamic systems, including natural phenomena, human behavior, and financial markets (Fernández-Díaz, 2024). Chaos theory explains inherent repetitions, patterns, self-organization, interconnections, and feedback loops within the visible randomness of complex, chaotic systems (Biswas et al., 2018). It paradoxically implies order and certainty because outcomes can be predicted based on initial conditions in a perfect system (Farazmand, 2003; Manera, 2021). Fractals are based on the principle that a simple process that goes through infinite iterations becomes complex. Fractals attempt to model complex processes by finding the simple processes underneath. Patterns, trends, and cycles can be discerned from the seemingly random events (Jahanmiri & Parker, 2022; Rosenbaum & Danesi, 2021; Werner, 2010).

Cellular automata are grids of cells whose states are determined by neighboring cells' states according to preset rules (Maeda & Sakama, 2007; Sottile & Padua, 2011). A conventional cellular automaton is local in that what happens in one cell is determined by what happens in the neighboring cells (Křehlík, 2023; Noble, 2021). In quantum mechanics, nonlocal "spooky actions" are observed in nature. Things that seem disconnected can be "entangled," influencing each other unknowingly (Boughn, 2022). Quantum cellular automata, including nonlocality, were created. Despite this, there is disagreement over whether nonlocal cellular automata support free will (Hameroff, 2012). An undetermined quantum event causing the brain to make a selection can be viewed as a physical event that caused the subject to make a selection (Schwartz et al., 2005). It is possible to view the process as a manifestation of free will. It is, however, arbitrary. Although the quantum event may not have been predetermined, the subject is alleged not to have controlled the decision (Vaidman, 2014).

The CDP considers chaos theory, fractals, and cellular automata fundamental to understanding systems. A system must have a degree of dynamically constrained variability to function correctly. Chaos is inherent to all systems and necessary for proper functioning, and fractals reflect the complexity of systems (Ilan, 2022a, 2023a, 2023b). The CDP does not account for feedback loops. A system seeks functionality by enabling a certain amount of disorder. Complex systems are unpredictable and require "freedom" and "unpredictability" to be efficient (Ilan, 2023b; Sigawi et al., 2023). According to the CDP, neurons and brains are unpredictable within constraints. These mechanisms are reflected in the "decisions" made by the brain. When a subject is "in control of a decision," it does not mean they are free to choose (Ilan, 2022a, 2023b).

II. Biologists frequently address free will. In the "nature versus nurture" debate, genetics and biology are compared with culture and environment as factors determining behavior (Jayaratne et al., 2009; Levitt, 2013). This debate has been transcended in most places in light of epigenetics. Genetics plays a role in shaping behavior and personality traits, which limits individual autonomy. There is a belief that all human behaviors can be attributed to genes, brains, and evolutionary histories (Briley & Tucker-Drob, 2014; Brown et al., 2011).

The variability of genes, proteins, and organs reflects the CDP and determines human behavior, providing the adaptability needed for function. The CDP views the constrained variability that characterizes genes, transcription, and biochemical reactions as fundamental for functioning biological systems and a driver for decisions and actions (Ilan & D YIM., 2019). It avoids interpreting genetic variability as human autonomy, which implies the personification of physical processes (Ilan, 2022a, 2023a, 2023b). The CDP accounts for all human actions that reflect an underlying physical variability in the brain and other organs. Subjects are continuously exposed to pressures and make decisions based on how they handle them (Sigawi et al., 2023).

III. Neuroscience studies how humans make decisions and act. Some claim it can imply that free will does not exist or is an illusion (Klemm, 2010). Libet's experiment, where subjects were asked to flick their wrists randomly while measuring the associated brain activity, resulted in an electrical signal called readiness potential. Half a second before subjects became aware of a conscious intention to move, unconscious brain activity began leading to movements, suggesting that the brain has unconsciously decided on the conscious mental act to do so (Libet et al., 1983). However, the onset of awareness of actions is accompanied by activity in the muscles underpinning the movement. Several additional studies questioned the conclusions about the causal nature of readiness potentials (Schurger et al., 2012). Compared to arbitrary decisions, a study found no early signs of decisions for deliberate ones (Lavazza, 2019; Maoz et al., 2019; Mudrik et al., 2022).

According to the CDP, a temporal trajectory is irrelevant to functionality. The inherent variability of the brain determines how it responds to stimuli and pressure from other organs in the body and the environment. It is an automatic response of the boundaries of variability that alters neuronal activity (Ilan, 2022a, 2023b).

The symptoms of Tourette syndrome and related tic disorders include involuntary movements and utterances (Swain & Leckman, 2005). Tics are not strictly involuntary and are classified as semivoluntary or involuntary. In some cases, they are voluntary responses to unwanted urges. It is sometimes possible for these subjects to suppress their tics for short periods (Mittal, 2020). Alien hand syndrome occurs when a limb moves unintentionally without the individual's will (Panikkath et al., 2014). There is an impairment in the premotor mechanism demonstrated temporarily by the appearance of the readiness potential on the scalp several hundred milliseconds before a spontaneous movement is overtly initiated. The supplementary motor complex on the medial surface of the frontal lobe activates before the primary motor cortex, possibly as part of a premovement preparatory process (Sarva et al., 2014). Among people younger than 65, frontotemporal dementia is the most common form of dementia that causes a loss of personality (Young et al., 2018). It is also possible for certain medications to cause personality changes. Genetics and early childhood events can significantly influence decisions (Cham et al., 2016; Likhar et al., 2022; Shikishima et al., 2015).

According to the CDP, these syndromes exhibit variability boundary malfunctions. They reflect a disruption of the brain-nerve-muscle connection due to mishandling of internal and external noises. A disturbed temporal association results from the dysfunction and is not its cause. Disease states are characterized by inappropriate variability, either too low or too high, which results in an inability to respond adequately (Ilan, 2023b; Ilan, 2022). CDP explains changes in personality as a result of dysfunctional boundaries. It implies that regaining some regulation of the function of the boundaries can assist in correcting diseased states.

IV. Psychological studies examined how people perceive free will and its role in assigning moral responsibility. The anthropological perspective of behavior infers a socially sanctioned reward and punishment system justified by free will (Shariff et al., 2014). Experimental psychology suggests that human perceptions of conscious control are illusions. People can have conscious will over behaviors they have not caused- conversely, for people to lack conscious will over behaviors they did cause. People tend to trust their introspections while distrusting others (Shepherd, 2015; Vonasch et al., 2018).

Under the CDP, the same law that governs all systems in nature also governs human behavior. It views "free will" as embedded within the response range determined by the boundaries that adapt to pressures. Human behavior reflects a machinery that dictates responses based on variability. Continuous pressures force humans to adapt continually. A lack of adaptation results in difficulties and unwanted feelings and responses. (Ilan, 2022a; Sigawi et al., 2023).

Studies have shown that randomness is not the only factor influencing free will (Joober & Karama, 2021). Free will can be mistaken for randomness because of its indeterminacy (Westen, 2005). An act is considered "free" when it involves a person opposing external forces, making a plan, or acting randomly. These "random" actions are impossible since participants' behavior betrays many patterns when they attempt to perform tasks randomly, such as generating random numbers (Chater et al., 2022; Curry et al., 2019). A superficial level of choice increased people's belief in free will. An adequate choice is essential, as too much or too little choice negatively affects belief (Feldman et al., 2014; Hornsby & Love, 2020; Liljenström, 2022).

CDP considers randomness a fundamental process in all systems, including human behaviors. Therefore, randomness is not a mistake in perception but a force that determines a behavior's outcome. "Random behavior" is normal as long as it remains within constraints. It reflects underlying variability in the brain and other organs and the degree of freedom in decision-making that arises from it. The CDP distinguishes subjective desire from a lack of desire at the level of brain neurons and molecules (Ilan, 2022a). Our insufficient knowledge of the processes governing consciousness does not justify unproven assumptions, which lead to misperception of desire or choice. Per the CDP, there is an "inherent level of choice" in how systems work. Human behavior and the decision process evolve from the variability machinery in the body's organs, and decisions always have a certain amount of randomness to them (Ilan, 2023a, 2023b).

According to some scientists, there is no such thing as free will (Lavazza, 2019). Based on determinism, the universe's history, including human decisions, was settled 13 billion years ago. It infers that prior events predetermine decisions, and indeterminism is just as incompatible with free will as determinism (Balaguer, 2019; Glattfelder & Glattfelder, 2019; Müller et al., 2019; Sandgren & Williamson, 2021). If decisions are not determined, they are not caused by anything, meaning they occur randomly (Liljenström, 2022). In some decisions, a subject considers the situation and comes to a clear conclusion with good reasons for making the choice. In torn decisions, the subject is unsure of what to do when confronted with options. It is adequate if the best option is chosen randomly. Selecting an option without being compelled can be considered free will, which may result from quantum probability (Abubakar et al., 2019; Cornwell et al., 2023; Liljenström, 2022; three contingencies of the debate, 2019).

According to the CDP, a degree of randomness is always present in the decision as it is the only way to make a good choice. Decisions are not based on free will but predetermined by how systems are designed to enable a degree of freedom (Ilan, 2023b). “Free will” is reflected by the CDP-based machinery's "free decision-making process." It applies to all decisions and reflects the brain's and neurons' variability. When there appears to be only one good choice, the boundaries limit the degree of variability based on the pressures. What seems like a random decision-making process is a valid functioning method (Ilan, 2022a, 2023b).

### CDP's Perspective on Superdeterminism

Superdeterminism is a theory based on hidden variables proposed by Bell (Julmi, 2019; Sen & Valentini, 2020). The peculiarities of quantum mechanics include the randomness of quantum events, reliance on human observation, and nonlocality, the ability of one measurement to influence another. The puzzle of nonlocality disappears if one assumes that nature is superdeterministic, including non-living systems and behaviors. In physics, hidden variables determine how experiments are conducted. Scientists might think they are choosing between two options, but they are not. As a result, freedom of choice implies incoherence and illogicality. In superdeterminism, there is no apparent randomness in quantum mechanics. Quantum mechanics is indeterministic because the outcomes are not determined. One can only predict probabilities for measurement outcomes in quantum mechanics, not the measurement outcomes themselves. An inability to predict the outcome of a quantum measurement results from missing information about hidden variables (Hossenfelder & Palmer, 2020; Keren & Schul, 2009; Kupczynski, 2023; Sen & Valentini, 2020; Vaidman, 2014).

Einstein doubted the existence of free will, believing specific causes must have specific, nonrandom effects (Landsman, 2020). This concept implies that the world is deterministic, so quantum mechanics must be deterministic. Superdeterminism eliminates measurement problems, nonlocality, and randomness, implying that measurement outcomes are more predictable than quantum mechanics claims (Gallego et al., 2013; Sen & Valentini, 2020). The hidden variables in quantum mechanics are not specified by superdeterminism. Their existence is decreed, and everything that happens is determined by what they specify, including people's decisions. As everything is made up of particles, invoking psychological causes does not affect the laws of physics (Schwartz et al., 2005).

The CDP implies that the same law governs all systems of the universe. Consequently, CDP-dependent functionality is found in every system reflecting "quantum variability" (Ilan, 2022a). It takes into account the hidden variables. Randomness controls functionality and is essential for proper function. Prediction is impossible due to the dynamic nature of the noisy universe. While there is no freedom to select, there is no determinism, as outcomes are always uncertain and undefined (Ilan, 2023b).

Many physicists believe that physics allows for free will (Vervoort, 2019). "Downward causation" implies that physical processes can cause "emergent" phenomena, particularly human desires and intentions, which affect our physical selves. A mathematical argument based on Bell's theorem of quantum nonlocality claims that subjects have free will because particles have it (Pernu, 2017; Hulswit, 2005).

According to the CDP, particles, and systems do not have intentions. They are functioning apparatuses. The embedded variability they characterize is not "free will," and they cannot select. The degree of freedom of systems is embedded in their design (Ilan, 2022a).

Philosophers refer to the gap between physical theories of consciousness and consciousness itself as an "explanatory gap." (Khrennikov, 2021) It is valid for consciousness and all human affairs. Humans are more than piles of interacting particles; they are intentional agents with psychological characteristics, mental states, and the capacity to make choices. Physics cannot explain love, desire, fear, hatred, justice, beauty, and morality. It entails that physics as a whole, rather than just quantum mechanics, is incomplete (Cheruvalath, 2007; Dupré, 2003; Thellman et al., 1962).

According to the CDP, feelings reflect brain activity and biochemical processes. Systems are characterized by variability, which enables them to make decisions under changing conditions. The results of this process are reflected in human behavior. Inherent freedom regulated by dynamic boundaries governs human actions and feelings (Sigawi et al., 2023).

Some argue that quantum mechanics can be re-configured as a cellular automaton to eliminate annoying features, such as its inability to predict exactly where an electron is (Norsen & Price, 2021; Wetterich, 2021). This model leads to superdeterminism, which implies the absence of free will. Wolfram, an expert on cellular automata, proposes that they can solve free will because of their "computational irreducibility." The cellular automata cannot be predicted in advance but are observed, and free will is compatible with this unpredictability (’t Hooft G., 2016; McDowell & Popa, 2009; Staubitz et al., 2017; t Hooft G., 2016). According to Conway and Kochen, quantum mechanics and relativity provide evidence for free will. When physicists measure the spin of particles, they can do so in dozens of ways, regardless of the preceding state of the universe. Particles' spins are not predetermined (Bao et al., 2020; Conway & Kochen, 2006; Koopmans & Youk, 2021). They suggested that physicists hold free will like the particles they are measuring. As experimenters possess a certain degree of freedom, so do the particles. Physicists and mathematicians use narrow, reductionistic frameworks to examine free will, and they equate free will with randomness and unpredictability (Lavazza, 2016; Tipler, 2014).

The CDP can interpret systems' unpredictable results as "freedom." A degree of freedom is built into the system, as it is not entirely deterministic. Systems are unpredictable and random by nature. They lack intentions; They, however, have mechanisms to help them maintain their functionality (Ilan, 2023a, 2023b).

### The CDP and the Attempt of "Having Both."

Choices may seem free because subjects cannot observe nature's laws in their fundamental form (LeBlanc, 2022). Particles operate within nature's rules in a way the senses cannot perceive, inferring an unknown force intervening in particle motion (Sparby et al., 2019). A subject's story about free will is valid based on their interpretation of human behavior. They cannot change anything that happens, but they can think and act as if they can. While classical chaos makes predicting the future impossible, it is deterministic. Even though quantum events are not deterministic, their apparent randomness cannot lead to willed actions. The theory of "having both" implies that some explanatory power vanishes at certain levels of reductionism, and some are gained (Allport, 1993; Chen, 2023; Lavazza, 2016; Mazzocchi, 2011; Santo & Gisin, 2022).

This recognition of "having both" is reflected in the CDP. It provides a fundamental platform for every system and enables a degree of freedom embedded in it. However, it does not allow intentional decisions. Psychology and society construct moral responsibility, not a physical principle, which underpins the CDP-dependent mechanisms that can be reflected in behavior and decisions.

Some claim that reducing everything to fundamental laws does not imply a reconstruction of the universe. Extrapolating properties from a few particles cannot explain a large and complex aggregate of elementary particles when new behaviors appear at each level of complexity. In this sense, free will is a psychological and neurological phenomenon rather than a physical one.

The term volitional decision-making is used to refer to free will. The decision-making process occurs at the neural network level, where sensory receptors and neurons gather coarse-grained information. They are not viewed as interventions in particle interactions (Menges, 2022; Oestreicher, 2007; Wang, 2008). Behavioral flexibility is considered an evolutionary superpower. Throughout life, subjects learn how to navigate the world, accumulating knowledge they can use in situations they have encountered. However, new scenarios require new solutions. It implies that the brain circuits offer action options based on their circuits' randomness (Holekamp et al., 2013; van Kesteren & Meeter, 2020; Ye & Li, 2021). In the two-stage model of free will, there is a small amount of randomness and some freedom at work, which influences the options that spring to mind (Holleman et al., 2020; James, 2010).

The CDP does not differentiate between systems based on size, providing a universal law that applies to all systems. Subatomic and atomic particles determine the outcome of neurons according to the same law and are reflected in human behavior (Ilan, 2023a). Brain variability facilitates cognitive adaptation to complex, unpredictable situations, reflecting CDP-based flexibility and adaptability. The ideas that "pop into the mind" are intuitive processes that subconsciously respond to the perturbations. The CDP describes an automatic model with an integral freedom reflected in neuronal variability. The "subconscious processes" reflect how systems are designed. The selection process appears intentional and is governed by neuronal and other brain processes characterized by inherent variability (Ilan, 2022a, 2023b; Sigawi et al., 2023).

Specialized brain systems rely on the noise created by networks of neurons. As subjects struggle to achieve their goals, their prefrontal and cingulate cortex send signals to the brain stem that regulate their behavior states. A cluster of neurons in the brainstem, the locus coeruleus, releases noradrenaline, raising alertness and vigilance and focusing attention on areas of uncertainty, signaling the cortex to act differently. A pattern of neurons that are active together encodes options for action in the cortex. These systems are flexible and adaptable to changing conditions (Aquino et al., 2023; Breton-Provencher et al., 2021; Dayan & Berridge, 2014; Faisal et al., 2008; Rolls, 2019). Circuits like these are noisy and change patterns in response to new information or at random. The noradrenaline released by the locus coeruleus contributes to the increased noise in the cortical areas. It resets the network, allowing it to retune into new patterns and expand the search for options in the brain (Liang et al., 2017; Ross & Bockstaele, 2020; Sohn et al., 2021). Some view this as a mechanism to develop creativity, similar to how evolution generates variation at random and then subjects it to selection. The same principle applies to antibody production in the immune system (Noble, 2021). These processes use a controlled source of randomness coupled with a selective filter. Errors and trials are more common. According to this theory, even when subjects use this noisy idea generator to expand their possibilities, their final decisions are still up to them (Avci et al., 2021; Liljenström, 2022).

The CDP asserts that the brain's and neurons' inherent variabilities are necessary to facilitate decision-making and adapt to changing environments correctly. This neuronal mechanism, like genetic and immune system variations, is not a method for improvement but for adaptation to ensure flexibility. Variability is not temporal but a means of optimizing functions under dynamic conditions (Ilan, 2022a, 2023b).

### Rest Waves in the Brain Reflect the CDP

The cerebral cortex is responsible for a person's behavioral functions. In neuroscience experiments, a stimulus is presented to the brain, and the brain is tested for response. "Resting waves" or "spontaneous waves" appear when there is no stimulus to the brain (Gu et al., 2021; Mehta et al., 2012). Free behavior produces resting waves anywhere in the cerebral cortex due to internal, spontaneous activity of the brain. People are unaware of the spontaneous waves (Livne et al., 2022; Tran et al., 2006; Wang et al., 2022). There are two components to rest waves, which work together (Broday-Dvir & Malach, 2020). The first is entirely random, and its source is biological noise. It is impossible to predict the outcome of each brain cell when grown in a dish because there are random vibrations inside a single cell, possibly even quantum ones (Capilla et al., 2022). As cells connect, they produce resting waves. The second type occurs when cells are connected, and their activity is slowed and synchronized in a way that suggests the expression of personality (Jedlicka, 2017). It is viewed as a mechanism for free behavior in the brain in a personalized way (Buskila et al., 2019; Wang et al., 2022). Neurons communicate through bundles of electrical pulses, and when there is something relevant to the function of certain types of cells, they increase activity; when there is nothing relevant, they decrease activity (Fields, 2020; Markett et al., 2018). Closing the eyes causes neurons in the visual system to change their firing rates synchronized. Synchronized waves in the cerebral cortex are characterized by a lack of awareness and by having personalized patterns for different subjects reflecting persons' knowledge and inclinations (Alejandre-García et al., 2022; Masquelier et al., 2011; Nowak et al., 2017).

This mechanism illustrates the CDP, where randomness leads to order through constraints on disorder. Systems consist of random elements and deterministic elements. Randomness is inherent in resting waves, but the structure of their connections limits their freedom. A system's "selection process" reflects its adaptability. In contrast to deterministic robots, the brain has both freedom and unique properties and the rest waves combine stochasticism and logic. The CDP argues that this process is essential and does not reflect errors, as proper brain function requires randomness. Combining randomness and synchronization can be interpreted as the system's inherent "freedom." Nevertheless, the CDP does not imply free will or creativity but flexibility (Ilan, 2019a, 2020a, 2020b, 2022a; Ilan, 2019).

An intrinsic, brain-wide spatiotemporal organization resulting from traveling waves is related to arousal. Fluctuations in arousal are associated with waves of activity that propagate slowly across the neocortex, cortex, thalamus, striatum, and cerebellum. These waves explain some aspects of spontaneous fMRI signal fluctuations (Mäki-Marttunen, 2023; Raut et al., 2021; Xiao et al., 2023). Neuronal activation is mandatory for consciously perceiving visual content. Contrary to rapid adaptation, the magnitude of neuronal activation drops over a short period while the visual stimulus and the conscious experience it evokes remain stable. Neuronal pattern profiles and their similarity distances are related to conscious perception rather than overall activation magnitude in the visual cortex (Raut et al., 2021). As a result of everyday experiences, cognitive processes fluctuate simultaneously. The retrospective behavioral sampling (RBS) method models cognitive fluctuations over time in response to stimulations across various cognitive dimensions. Cognitive fluctuations are replicable across distinct cognitive dimensions, revealing a stimulus-driven experience shared among individuals. Different stimuli do not alter idiosyncratic response patterns, suggesting that RBS identifies individual differences independent of stimulus (Broday-Dvir et al., 2023).

The data exemplifies the CDP by demonstrating that brain fluctuations, altered neuronal profiles, and RBS fluctuations are required for proper brain functionality.

### Future Challenges: Directions and Implications for Using CDP-Based Artificial Intelligence to Overcome Malfunctions

Using the CDP to understand free will can improve therapeutic approaches. According to the CDP, the cognitive processes reflect the dynamic boundaries. The inherent variability of the brain facilitates decision-making. Adaptation mechanisms require randomness and dynamicity in decision-making under known and unknown conditions. A CDP definition of creativity holds that the ability to make decisions under unfamiliar conditions reflects the dynamic boundaries of neuronal variability, enabling cognitive flexibility. The degrees of variability in neuronal activity are crucial to setting up a range of choices. According to the B = F formula, the dynamic freedom of the degree of variability provides a range of choices for the individual to make. This range reflects the yet-to-be-defined apparatus or mechanism by which a final decision is made that is adaptable to noisy perturbations. Decision-making processes are enabled by the boundaries of the constraints of choice, enabling selection within a predefined dynamic range of options. Per the CDP, there is no memory in the direct sense of the word; however, past events are embedded in the function of the boundaries, allowing individualization of decision-making across subjects (Ilan, 2022a; Sigawi et al., 2023).

In the CDP, dysfunctions of the boundaries are reflected by too high or too low variability, which can lead to disease conditions (Ilan, 2023b). As constrained variability is required for proper function, a lack of constraints indicates a system's faults. A healthy function and the ability to make productive decisions require a certain degree of variability. Abnormal free will and excessive freedom reflect high noise levels, affecting decision-making (Sigawi et al., 2023).

The incorporation of CDP-based principles into systems can assist in overcoming their dysfunctions. It involves incorporating CDPs into therapeutic regimens and psychological models (Brandman et al., 2022). The CDP-based second-generation AI system incorporates variables into interventions in a three-level model (Brandman et al., 2022; Hurvitz & Ilan, 2023; Ilan, 2020c, 2020d; Ilan, 2022; Brandman et al., 2022).

The first level of CDP-based algorithms is open-loop, independent of outcome, and introduces a degree of variability into the processes. This variability has a predefined range. The second level of the platform is based on a closed-loop algorithm that continuously changes the degree of variability according to the required outcome. It provides an individualized system that continuously adjusts the level of variability of the intervention to the outcome. A third level of the platform comprises individualized variability signatures that reflect proper functionality and are constantly quantified and incorporated into the algorithm to optimize the outcome (Ilan, 2020c).

The digital pill is an example of using a CDP-based second-generation artificial intelligence system to improve the response to chronic medications, overcome tolerance, and reduce side effects (Adar et al., 2023; Azmanov et al., 2021, 2022; Gelman et al., 2020, 2023; Hurvitz et al., 2021, 2022; Ilan, 2019e, 2020d, 2021; Ilan & Spigelman, 2020; Isahy & Ilan, 2021; Ishay et al., 2021a, 2021b; Kenig & Ilan, 2019; Kenig et al., 2021; Kessler et al., 2020; Khoury & Ilan, 2019, 2021; Kolben et al., 2021, 2023; Potruch et al., 2020; Sigawi & Ilan, 2023). Incorporating CDP principles to improve outcomes can benefit psychological therapies and interventions to improve decision processes. Platforms based on CDPs can enhance personal development, wellness, aging, and mental health.

## Conclusions

The author Isaac Bashevis Singer expressed it by saying: "We must believe in free will – we have no choice." (Gelman et al., 2023) In his book: "Life Without Free Will," Sapolsky writes that there is a chain of biological and environmental causes that lead to every thought, action, and experience, starting with the moment a neuron is activated and lasting throughout history. "Nowhere in this continuum is there a place into which free will can be crammed." Intentions are so tangible that people confuse them with freedom. When one analyzes the source of that intention, it is clear that it comes from everything that makes a subject who he is, over which he has no control (Wertenbroch et al., 2008).

CDP is a universal law that applies to all systems in nature. While people may believe in free will, science may contradict it. The CDP provides a system for understanding free will and overcoming challenges in systems dysfunction related to free will. Future studies are designed to explore using CDP-based systems to overcome some challenges in dissecting the mechanism associated with free will and improve the use of CDP-based systems for improving performance.

## Data Availability

NA.

## Abbreviations

CDP: Constrained disorder principle
HRV: Heart rate variability
AI: Artificial intelligence
